# Refractory marginal zone lymphoma uncovers activated phosphoinositide 3-kinase delta syndrome type 1 (APDS1)

**DOI:** 10.1016/j.jacig.2025.100503

**Published:** 2025-05-26

**Authors:** Alex Wonnaparhown, Matthew Farley, Holly Miller, Charlotte Cunningham-Rundles, Catherine Freeman, Jacqueline Squire, Talal Hilal, Allison Rosenthal

**Affiliations:** a Division of Allergy, Asthma, and Clinical Immunology, Mayo Clinic, Phoenix, Ariz; e Division of Hematology and Medical Oncology, Mayo Clinic, Phoenix, Ariz; b Center for Cancer and Blood Disorders, Phoenix Children's/Mayo Clinic Arizona, Phoenix, Ariz; c Division of Allergy and Clinical Immunology, Department of Medicine and Department of Pediatrics, Icahn School of Medicine at Mount Sinai, New York, NY; d Division of Allergy, Asthma, and Clinical Immunology, Mayo Clinic, Jacksonville, Fla

**Keywords:** Marginal zone lymphoma, primary immunodeficiency, activated phosphoinositide 3-kinase delta syndrome, genomics, case report

## Abstract

Activated PI3K delta syndrome is a rare primary combined immunodeficiency that can present with lymphoma. Genetic testing of patients with atypical lymphoma may reveal an underlying immunodeficiency and improve clinical outcomes.

## Case report

A 27-year-old man of Middle Eastern and Czechoslovakian descent was referred for refractory marginal zone lymphoma (MZL) and a history of dysphagia. He was incidentally diagnosed with MZL via stomach biopsy during an endoscopy to evaluate eosinophilic esophagitis. Testing for *Helicobacter pylori* yielded a negative result. A positron emission tomography–computed tomography (PET-CT) scan showed focal uptake in the rectum, a 16-cm spleen, and nodular parotid glands. Despite radiation therapy, subsequent PET-CT scans showed increasing neck lymphadenopathy and persistent hypermetabolism at the rectosigmoid junction, with persistent MZL on repeat rectal biopsy. The patient’s medical history was otherwise notable for splenomegaly, recurrent thrombocytopenia, ileocolic intussusception, elevated IgG level, parotid gland lesions, pancytopenia, iron deficiency, and eosinophilic esophagitis. His infection history consisted of 2 or 3 episodes of bronchitis or otitis media per year in childhood. However, he did not report any severe infections in the past 10 years. He denied a family history of immunodeficiency.

His physical examination showed splenomegaly, alopecia areata, and 2 palpable right posterior cervical lymph nodes. His laboratory results are shown in Table I. His pneumococcal titers were protective (≥1.3 μg/mL) for 8 of 22 serotypes, decreasing to 5 of 22 serotypes (4 weeks and 5 days after vaccination with the PPSV23 vaccine). His last Tdap (tetanus toxoid, reduced diphtheria toxoid, and acellular pertussis vaccine, absorbed) booster was administered 1 year and 8 months before lymphocyte proliferation testing.

**Table I:** Laboratory test results

Blood test	Result	Reference range
Hemoglobin level (g/dL)	**12.7 (low)**	13.2-16.6
Hematocrit (%)	**40.5 (low)**	38.3-48.6
Platelet level (×10^9^/L)	**83 (low)**	135-317
Leukocyte level (×10^9^/L)	**2.4 (low)**	3.4-9.6
Neutrophil level (×10^9^/L)	1.63	1.56-6.45
Lymphocyte level (×10^9^/L)	**0.430 (low)**	0.95-3.07
Monocyte level (×10^9^/L)	**0.160 (low)**	0.26-0.81
Eosinophil level (×10^9^/L)	0.130	0.03-0.48
Basophil level (×10^9^/L)	0.030	0.01-0.08
Hepatitis C antibody	Negative	
Hepatitis B surface and core antibody	Negative	
EBV DNA	Negative	
CMV DNA	Negative	
BK virus DNA (in urine)	**8620 (high)**	Undetectable
IgG level (mg/dL)	**2139 (high)**	767-1590
IgM level (mg/dL)	217	37-286
IgA level (mg/dL)	**514 (high)**	61-356
IgE level (kU/L)	47.9	≤214
Tetanus antibody level	0.44 IU/mL	
Diphtheria antibody level	**0.02 IU/mL (low)**	
NK cells (%)	24	5-26
NK cell level (cells/μL)	109	84-724
Proliferation to *Candida* (% of CD45 cells)	**0.9 (low)**	≥5.7
Proliferation to *Candida* (% of CD3 cells)	**1.1 (low)**	≥3.0
Proliferation to tetanus (% of CD45 cells)	**0.0 (low)**	≥5.2
Proliferation to tetanus (% of CD3 cells)	**0.0 (low)**	≥3.3
Proliferation to PWM (% of CD45 cells)	10.6	≥4.5
Proliferation to PWM (% of CD3 cells)	14.1	≥3.5
Proliferation to PWM (% of CD19 cells)	15.7	≥3.9
Proliferation to PHA (% of CD45 cells)	**38.5 (low)**	≥49.9
Proliferation to PHA (% of CD3 cells)	**52.8 (low)**	≥58.5
CD19^+^ cells (% of total lymphocytes)	15.7	2.8-17.4
CD20^+^ cells (% of total lymphocytes)	**17.0 (high)**	3.2-16.8
CD27^+^IgM^+^IgD^+^ cells (% of CD19^+^ B cells)	**1.4 (low)**	1.7-29.3
IgM^+^ cells (% of CD19^+^ B cells)	**83.9 (high)**	26.0-78.0
CD38^+^IgM^+^cells (% of CD19^+^ B cells)	**76.8 (high)**	1.2-50.7
CD4 T cells (/μL)	**128 (low)**	365-1437
CD8 T cells (/μL)	**99 (low)**	199-846
CD4^+^CD45^+^CD31^+^ recent thymic emigrants (%)	**4.1 (low)**	6.4-41.7
Level of CD4^+^CD45^+^CD31^+^ recent thymic emigrants (/μL)	**6.8 (low)**	42.0-399.0

Boldface indicates an abnormal result.

*CMV*, Cytomegalovirus; *NK*, natural killer; *PWM*, pokeweed mitogen.

His updated PET-CT scan showed stage IV progression of cervical lymphadenopathy, continued splenomegaly (17.5 cm), and worsening focal hypermetabolism in the rectosigmoid. Biopsy of the rectal mass revealed mucosa-associated lymphoid tissue lymphoma with κ light chain–restricted B cells, positivity for clonal immunoglobulin gene rearrangement, and clonal T-cell receptor rearrangement. Targeted next-generation blood DNA testing revealed the heterozygous pathogenic variant c.3061G>A (p.Glu1021Lys) in *PIK3CD* with an allele frequency of 42% to 52%. This variant was not present in his parents or sister.

**Fig 1:**
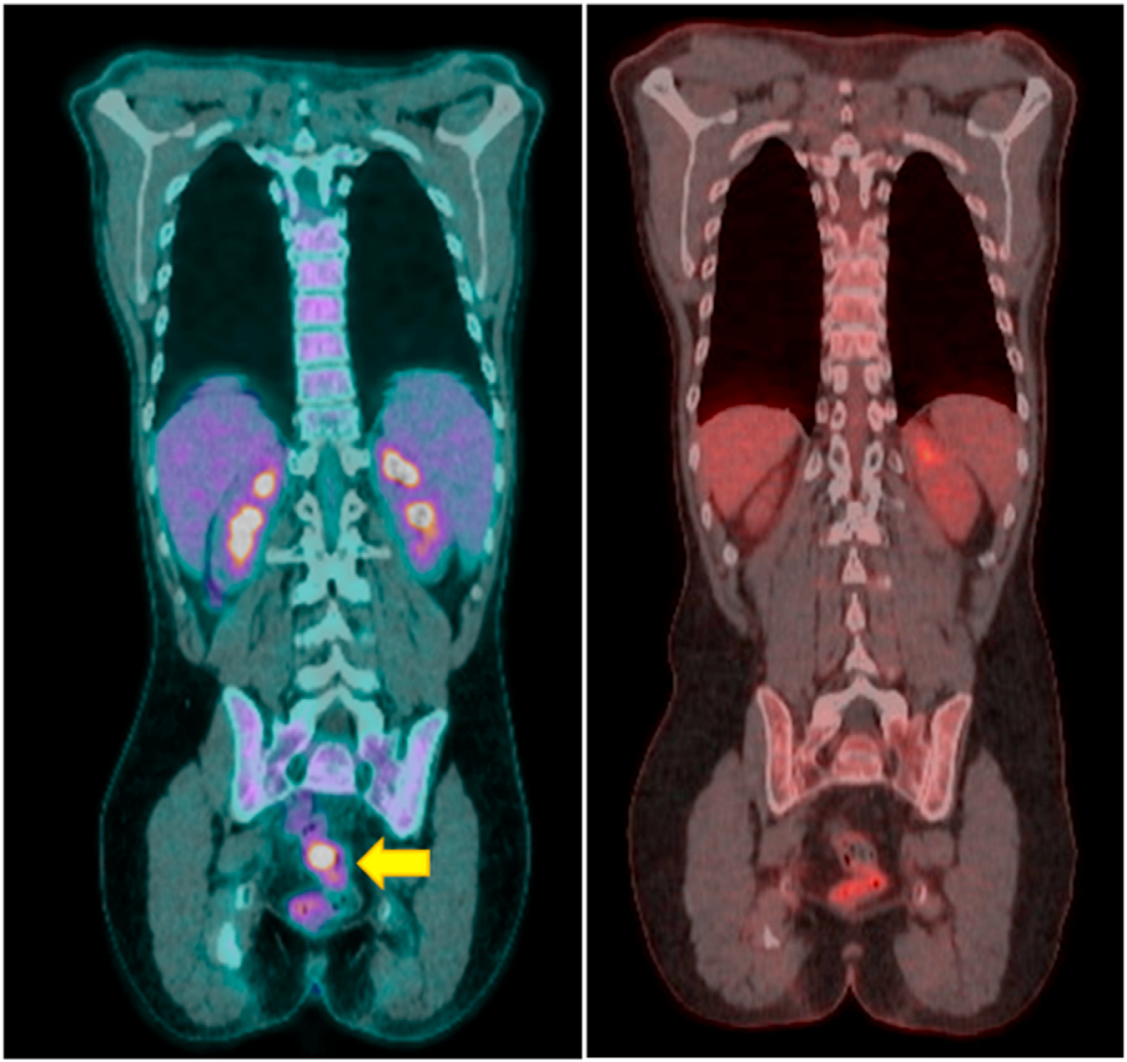
PET-CT scans before and after treatment with rituximab. Before (*left*) and after (*right*) treatment with rituximab and sirolimus shows resolved rectal uptake and decreased spleen size.

He completed 4 weekly doses of rituximab, 375 mg/m^2^, with a significant lymphoma response. Repeat PET-CT showed resolved cervical lymphadenopathy, absence of gastrointestinal tract abnormalities, and decreased spleen size (to 14.5 cm [Fig 1]). He began taking sirolimus, 2 mg/m^2^ per day, and tolerated this dose well, with a goal trough of 5 to 15 ng/mL. Despite his elevated IgG level, he was also given intravenous immunoglobulin, 0.5 g/kg, because his laboratory test results suggested a functional antibody deficiency based on poor vaccine response; however, his clinical history did not suggest a history of serious recurrent viral or bacterial sinopulmonary infections. He continues taking trimethoprim/sulfamethoxazole, single-strength daily, for *Pneumocystis jiroveci* pneumonia prophylaxis. He started taking valacyclovir, 500 mg twice per day, which was then discontinued after discussion with the patient and absence of a severe viral infection history. Administration of fluconazole 200 mg daily, was cautiously started for coccidioidomycosis prophylaxis but was discontinued owing to rising transaminase levels after only 2 weeks. The patient was encouraged to receive inactivated vaccines and advised to avoid live vaccines. He initially desired to continue taking sirolimus because of his clinical stability, but he has now agreed to transition to leniolisib with a planned 2- to 4-week sirolimus washout period. He underwent evaluation for hematopoietic stem cell transplantation with the National Institutes of Health, but he does not have a matched donor and is not slated for a transplant at this time.

## Discussion and conclusion

Activated phosphoinositide 3-kinase delta syndrome (APDS) is now recognized as a primary combined immunodeficiency caused by gain-of-function mutations in *PIK3CD* (APDS type 1 [APDS1] or loss-of-function mutations in *PIK3R1* (APDS type 2 [APDS2]) that lead to hyperactivation of the kinase PI3Kδ, which contains the subunits p110δ (catalytic) and p85α (regulatory). Inappropriate hyperactivation of PI3Kδ and the mammalian target of rapamycin (mTOR) pathway lead to overactive and impaired lymphocyte proliferation, T-cell senescence and death, increased regulatory T-cell count, increased transitional B-cell count, and impaired class switch recombination and somatic hypermutation.^1^ APDS is typically inherited in an autosomal dominant pattern, but *de novo* mutations have been reported. Characteristic features include antibody deficiency, defective T-cell function, lymphoproliferation, increased transitional B-cell and plasmablast counts, and increased risk of B-cell lymphoma (Table II).^2^ The prevalence of malignancy is as high as 30% in APDS1, with Hodgkin lymphoma and diffuse large B-cell lymphoma being the most prevalent; however, 4 cases of MZL have been reported in patients with APDS1 (and 1 case in a patient with APDS2), with a median age of 15 years at diagnosis (range 13-18 years).^3^

**Table II:** Clinical and immune characteristics of APDS1

• Recurrent infections (especially pneumonia and bronchiectasis) • Chronic viral infections (EBV, HPV, VZV, or CMV) • Hepatosplenomegaly • Autoimmune disease • Enteropathy • Glomerulonephritis • Cytopenia • Developmental delay or short stature • Antibody deficiency (low IgG, IgA, and/or IgM levels) • Reduced or absent responses to pneumococcal or tetanus vaccine • Decreased numbers of CD19^+^, naive, marginal zone (CD27^+^IgM^+^IgD^+^), and/or switched/unswitched B cells • Increased numbers of transitional CD38^+^IgM^+^ B cells • Decreased numbers of naive CD45RA^+^ T cells • Increased numbers of central and effector memory CD4^+^CD45RA^–^ T cells • Increased numbers of effector memory CD8^+^CD45RA^–^ T cells

In patients with lymphoma, these findings may prompt additional genetic evaluation for underlying APDS1.

*CMV*, Cytomegalovirus; *HPV*, human papillomavirus; *VZV*, varicella-zoster virus.

As of 2024, 193 cases of APDS1 had been reported in the literature, with a median patient age of 13 years.^4^ E1021K is the most commonly reported pathogenic variant; it is located in the C-lobe of the kinase domain and results in enhanced kinase activity of the p110δ subunit.^5^ The majority of patients with APDS typically present with recurrent and severe infections as early as during the first year of life.^2^^,^^4^ A systematic review of the cases of a total of 256 patients with APDS1 and patients with APDS2 suggests that survival rate decreases as patients age, with a lymphoma mortality rate of 47.6%.^4^ In a cohort of 53 patients with APDS1, including 50 patients with the E1021K mutation, clinical presentation ranged from minimally symptomatic in adulthood to early death.^6^

Treatment of APDS involves antimicrobial prophylaxis, IgG replacement therapy, and either sirolimus (inhibition of mTOR downstream of phosphoinositide 3-kinase delta [PI3Kδ]) or leniolisib (a selective PI3Kδ inhibitor). Leniolisib was US Food and Drug Administration–approved for APDS after a phase 3 trial in 2023 showed improvement of naive B cells and decreased splenomegaly with minimal adverse events.^7^ The open-label extension study of leniolisib in 37 patients showed outcomes lasting for up to 5 years.^8^ None of the patients with prior lymphoma had relapses of lymphoma; however, longer follow-up is essential. Antibiotic prophylaxis is typically performed with trimethoprim/sulfamethoxazole. Antiviral prophylaxis can help those with history of severe and/or recurrent Herpesviridae-related infections. Coccidioidomycosis prophylaxis can also be considered in endemic areas. Hematopoietic stem cell transplantation has been successful, although mTOR inhibitors were possibly associated with higher rates of graft failure if used in the first year after transplantation.^9^

In summary, APDS1 is a primary combined immunodeficiency that is caused by gain-of-function mutations in *PIK3CD*; it induces hyperactivation of PI3Kδ and leads to immunodeficiency, systemic lymphoproliferative complications, autoimmunity, and malignancy. Definitive diagnosis is made with genetic testing and is crucial for prompt and appropriate treatment to prevent long-term complications. Awareness of APDS as well as collaboration between hematology/oncology and allergy/immunology for timely genetic testing for underlying primary immunodeficiency in atypical lymphoma can improve clinical outcomes. Future consideration of genetic screening in all patients with mucosa-associated lymphoid tissue lymphoma may uncover a higher underlying prevalence of APDS and other primary immunodeficiency.

## Disclosure statement

Supported in part by generous Paula and Roger Riney Foundation funding and in part by generous funding from additional philanthropic donations to the 10.13039/100000871Mayo Clinic, as well as by the 10.13039/100000054National Cancer Institute (grant U01CA271410). Publication fees are supported by the 10.13039/100000871Mayo Clinic Hematological Malignancies Program. The content is solely the responsibility of the authors and does not necessarily represent the official views of the National Institutes of Health.

Disclosure of potential conflict of interest: The authors declare that they have no relevant conflicts of interest.

## References

[1] Lucas C.L., Chandra A., Nejentsev S., Condliffe A.M., Okkenhaug K. (2016). PI3Kdelta and primary immunodeficiencies. Nat Rev Immunol.

[2] Thouenon R., Moreno-Corona N., Poggi L., Durandy A., Kracker S. (2021). Activated PI3Kinase delta syndrome-a multifaceted disease. Front Pediatr.

[3] Durandy A., Kracker S. (2020). Increased activation of PI3 kinase-δ predisposes to B-cell lymphoma. Blood.

[4] Hanson J., Bonnen P.E. (2024). Systematic review of mortality and survival rates for APDS. Clin Exp Med.

[5] Singh A., Joshi V., Jindal A.K., Mathew B., Rawat A. (2020). An updated review on activated PI3 kinase delta syndrome (APDS). Genes Dis.

[6] Coulter T.I., Chandra A., Bacon C.M., Babar J., Curtis J., Screaton N. (2017). Clinical spectrum and features of activated phosphoinositide 3-kinase delta syndrome: a large patient cohort study. J Allergy Clin Immunol.

[7] Rao V.K., Webster S., Sediva A., Plebani A., Schuetz C., Shcherbina A. (2023). A randomized, placebo-controlled phase 3 trial of the PI3Kdelta inhibitor leniolisib for activated PI3Kdelta syndrome. Blood.

[8] Rao V.K., Kulm E., Sediva A., Plebani A., Schuetz C., Shcherbina A. (2024). Interim analysis: open-label extension study of leniolisib for patients with APDS. J Allergy Clin Immunol.

[9] Dimitrova D., Nademi Z., Maccari M.E., Ehl S., Uzel G., Tomoda T. (2022). International retrospective study of allogeneic hematopoietic cell transplantation for activated PI3K-delta syndrome. J Allergy Clin Immunol.

